# Fluorescent, multifunctional anti-counterfeiting, fast response electrophoretic display based on TiO_2_/CsPbBr_3_ composite particles

**DOI:** 10.1038/s41377-024-01526-x

**Published:** 2024-08-20

**Authors:** Guangyou Liu, Xinzao Wu, Feng Xiong, Jinglan Yang, Yunhe Liu, Jie Liu, Zhuohang Li, Zong Qin, Shaozhi Deng, Bo-Ru Yang

**Affiliations:** State Key Laboratory of Opto-electronic Materials and Technology, Guangdong Province Key Laboratory of Display Materials and Technologies, School of Electronics and Information Technology, Sun Yat-Sen University, Guangzhou, 510006 China

**Keywords:** Quantum dots, Nanoparticles

## Abstract

Traditional optical anti-counterfeiting (AC) is achieved by static printed images, which makes them susceptible to lower levels of security and easier replication. Therefore, it is essential to develop AC device with dynamic modulation for higher security. Electrophoretic display (EPD) has the advantages of low power consumption, high ambient contrast ratio, and capability of showing dynamic images which is suitable for dynamic AC applications. Herein, we prepared a dynamical AC device based on a fluorescent EPD, and achieving the image switch between black, white, and green fluorescence states under the dual-mode driving (electronic field and UV light). We loaded perovskite quantum dots (CsPbBr_3_) onto the TiO_2_ particles and further prepared fluorescent electrophoretic particles TiO_2_/CsPbBr_3_-3-PLMA (TiO/CPB-3) by grafting and polymerizing method. In addition, we fabricated the AC devices based on the fluorescent EPD, which exhibits the multifunctional AC, where the fluorescent EPD has a fast response time of 350 ms, a high contrast ratio of 17, and bright green fluorescence. This prototype demonstrates a new way for future dynamic AC and identification.

## Abstract (web summary)

We fabricated a multifunctional anti-counterfeiting (AC) device based on fluorescent electrophoretic particles, which can achieve dynamic AC display by switching the electric field and UV light.

## Graphical abstract

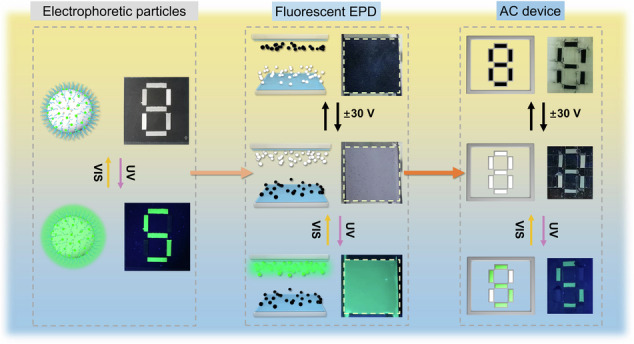

## Introduction

Anti-counterfeiting (AC) technologies can effectively prevent counterfeit products and fakes^1,2^. Currently, AC technologies can be widely used in the product labels, passports, secret documents, etc.^3,4^. In addition, the market of global AC market was $ 135.73 billion in 2022, with expected annual growth rate of 12.1% from 2023 to 2030^5,6^. In recent years, most of AC technologies are based on watermarks, dynamic two-dimensional code, thermal response, and luminescence^7–9^. Compared with other AC technologies, optical AC devices possess good display performance, convenience, and fast response. Many optical materials have been developed for optical AC devices, including quantum dots, upconversion materials, electroluminescence, and photonic crystals^10–13^. These optical materials and devices can be excited under ultraviolet (UV) light, near-infrared, electric field, and other types^14,15^. However, single-functional AC technologies are less secure and easily duplicated. Therefore, it is essential to develop the AC device with dynamic display and multi-function.

Electrophoretic display (EPD) has the advantages of low power consumption, dynamically switched the image, and high ambient contrast ratio, which is suitable for dynamic AC technology. In addition, EPD is a reflective display that control the movement of charged black and white electrophoretic particles under the electric field, and then present the images^16,17^. Due to the low power consumption and eye protection, EPD has been widely used in e-books, shelf labels, bus stops, smart windows, and so on^18–22^. However, the commercial EPD can only be used under visible light sources (sunlight, lamp, front light source), and the display performance of EPD would be highly dependent on the light intensity of visible light, existing light source dependence, which hinders the EPD application under a dark environment and non-visible light. Fluorescent EPD could exhibit the patterns under UV light sources, which extends the application scenario of traditional electrophoretic display under ultraviolet light. Therefore, it is meaningful to introduce the fluorescent electrophoretic particles into EPD, which effectively solve visible light dependence of traditional EPD. By combining the reflection and emission modes, the fluorescent EPD has the advantage of dual driving mode, allowing it to be used in both dark and visible light. Unfortunately, the application of AC device based on fluorescent electrophoretic particles has rarely been reported.

Currently, the fluorescent electrophoretic particles can be introduced into fluorescent EPD, which can switch between reflection and emission modes under UV light switching. Meng et al.^23^ coated fluorescent dyes on the electrophoretic particles and fabricated the first fluorescent EPD with emissive and reflective display. Recently, Hong et al.^24^ designed a dual-mode electrophoretic display device based on the BaMgAl_10_O_17_:Eu^2+^, Mn^2+^ particles, which can switch between white and blue states under UV light on/off. However, response time and luminescent intensity of the fluorescent EPD need to be further improved. Therefore, to develop luminescent intensity of the EPD, it is feasible to introduce the PQD (perovskite quantum dot) into EPD. Generally, the inorganic metal halide perovskite nanocrystals and PQD (CsPbX_3_, X = Cl^−^, Br^−^, or I^−^) are widely used in optoelectronic devices due to its high luminescent, adjustable emission peaks, and low fabrication cost^25–29^. Since charged ligands load on the surface of PQDs, which can be electrophoretic deposited on the patterned electrode with good uniformity and orientation under the electric field^30–32^. Therefore, it is effectively to orient drive the QDs under the electric field, which can be applied in fluorescent EPD. Jin et al.^33^ first prepared homogeneous and high-density perovskite thin films (MAPbBr_3_) by using electrophoretic deposition on a PEDOT: PSS coated ITO electrode, which exhibited a high quantum efficiency of 80-90%. Recently, Fulari^34^ and Li^35^ fabricated photodetectors based on PQDs film by electrophoretic deposition, respectively. Therefore, based on the above research work in electrophoretic deposition, it is feasible to introduce PQDs as fluorescent electrophoretic particles into fluorescent EPD, and achieving the EPD with reversible driving and multifunctional display.

Herein, we loaded CsPbBr_3_ (CPB) onto the electrophoretic particle TiO_2_ (TiO_2_/CsPbBr_3_-3 (T/C-3), the mass of TiO_2_ is 300 mg in CsPbBr_3_ solution) and prepared the fluorescent electrophoretic particle, which showed a white color under ambient light, and a green color under UV light. Combining CPB with TiO_2_ particles can obtain the fluorescent electrophoretic particles with high charge, whiteness, and strong fluorescence intensity, which can make the fluorescent electrophoretic particles drive fast under the electric field. Besides, the long distance of the TiO_2_/CsPbBr_3_-3 between the green state and white state in CIE diagram, indicating that the fluorescent electrophoretic particles have a good potential application in AC devices. Then, we fabricated the fluorescent EPD based on TiO_2_/CsPbBr_3_-3-PLMA (TiO/CPB-3), which has a fast response time of 350 ms and high contrast ratio of 17. In addition, the fluorescent EPD achieves a multifunctional display by the switching of electric field and UV light. Under the switching of the electric field, the EPD shows black-and-white state switching under visual light. Besides, the device achieves the green and white state switching under UV light switching, exhibiting excellent AC optical performance. Combining the advantages of self-luminous and reflective display, the fluorescent EPD based on fluorescent electrophoretic particles TiO/CPB-3 can work under the different ambient light illuminance environments. Final, we fabricated the AC devices based on the fluorescent EPD, which exhibits the multifunctional anti-counterfeiting. This work demonstrated that fluorescent EPD has a fast response, dynamically switch image and bright luminescence, providing potential in multifunctional AC applications.

## Results

**Fig. 1:**
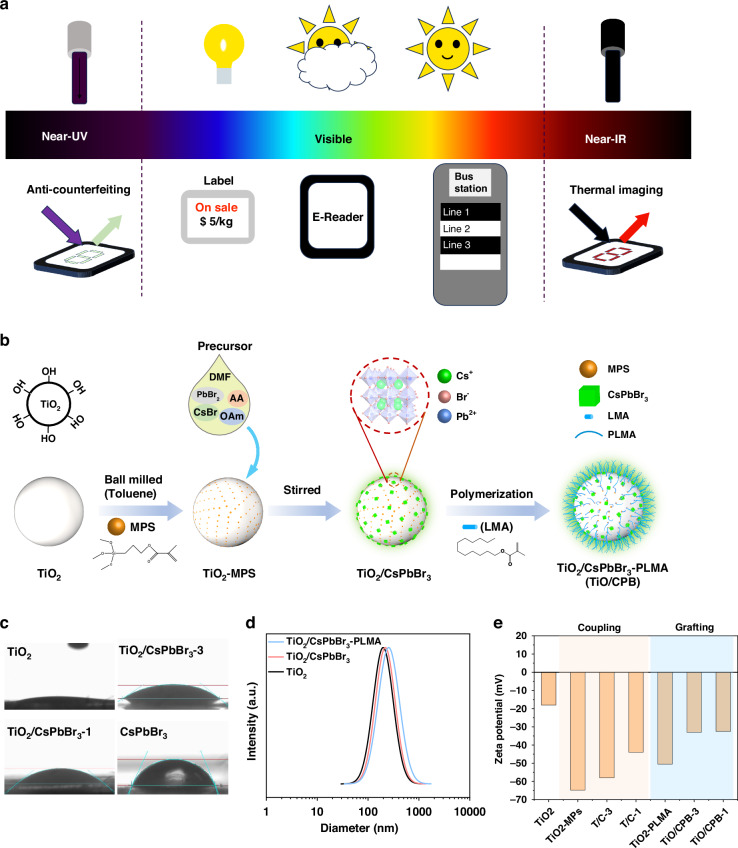
The application scenarios and preparation of the fluorescent electrophoretic particles. **a** Application scenarios of multifunctional e-paper in different wavelength. **b** Illustration of preparation of fluorescent electrophoretic particles. **c** The contact angle of the TiO_2_, TiO_2_/CsPbBr_3_-1, TiO_2_/CsPbBr_3_-3 and CsPbBr_3_. **d** DLS spectra of TiO_2_, TiO_2_/CsPbBr_3_ and TiO_2_/CsPbBr_3_-PLMA. **e** The zeta potential of the fluorescent electrophoretic particles

Currently, most EPDs are widely applied in visible light, such as sunlight, lamps, and backlight, which suffer from light source dependence. EPDs can be used in shelf labeling, e-books, and bus stops under visible light, as shown in Fig. 1a. While the fluorescent EPD can be applied in AC devices under UV light, the infrared EPD can be applied to thermal images under infrared light. To extend the application of EPD, we have prepared the fluorescent electrophoretic particles by loading the CsPbBr_3_ onto the TiO_2_ particles, exhibited in Fig. 1b. The surface of TiO_2_ particles contains lots of hydroxyl functional groups which are benefited for the further surface treatment and graft polymerization. Firstly, the TiO_2_ particles were ball-milled with a silane coupling agent to obtain TiO_2_-MPS, and the C = C bonds in MPS facilitated the polymerization reaction. Then, the quantum dot precursor solution was poured into the toluene solvent with dispersed TiO_2_. Since the toluene solution is non-polar solvent, the perovskite CsPbBr_3_ would precipitate on the surface of TiO_2_ particles (TiO_2_/CsPbBr_3_). Finally, we polymerized the TiO_2_/CsPbBr_3_ particles with the polymer monomer LMA to obtain fluorescent electrophoretic particles TiO_2_/CsPbBr_3_-PLMA. Due to the low thermal stability of the QD CsPbBr_3_, we used the ABVN as the initiator of the LMA, which initiated the polymerization reaction at the low temperature of 40 °C^36,37^.

The hydrophilicity of fluorescent electrophoretic particles was tested by the contact angle, as shown in Fig. 1c. The TiO_2_ particles exhibit strong hydrophilicity with an angle lower than 10° due to their hydroxyl-rich functional groups. In addition, the contact angle of QDs is 50° due to the oleylamine (OAm) and acrylate long-chain ligands. Compared with the pure TiO_2_, the TiO_2_/CsPbBr_3_-3 exhibits a more hydrophobicity with contact angle of 40°, and the hydrophobic particles are beneficial to the dispersion in non-polar solvents. To measure the size of the particles, we tested the DLS spectra, as shown in Fig. 1d. The size of the electrophoretic particles would affect the light scattering. If the particle size is too small, the light penetrates the particles, and devices exhibit a transparent state. On the contrary, if the particle size is too large, the particles would stack unevenly, resulting in the deteriorating display performance of the device. The particle size of TiO_2_ is concentrated at 200 nm. After silane coupling and loading QD, the size of the fluorescent electrophoretic particles increases to 230 nm. After the polymerization, the size of the fluorescent electrophoretic particles increases to 250 nm, which is suitable for reflective display.

The zeta potential (*ζ*) of fluorescent electrophoretic particles would influence the performance of EPD. The zeta potential of pure TiO_2_ is negatively charged (-15 mV) due to the hydroxyl group, as shown in Fig. 1e. After grafting the silane, the hydroxyl and ester groups would increase the negative charge on the TiO_2_ particles to -65 mV. Due to the coating of QDs, the surfactant on the QDs would reduce the zeta potential of TiO_2_/CsPbBr_3_-3 to -60 mV. Besides, the polymer layer would hinder the charged behavior between the particles and the CCA, thus decreasing the potential to -30 mV. The photograph of TiO_2_, TiO_2_/CsPbBr_3_, and TiO_2_/CsPbBr_3_-PLMA under visible and UV light are shown in Figure S1. All three powders show a white color under ambient light. At the same time, TiO_2_ would absorb the UV light and exhibit the black state. The fluorescent electrophoretic particles exhibit green light under the UV light, which has potential applications in AC devices.

**Fig. 2:**
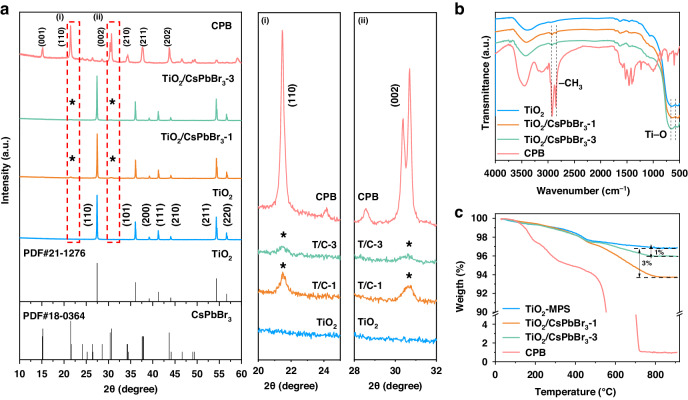
Characterization of fluorescent electrophoretic particles. **a** The XRD spectrum of the TiO_2_, TiO_2_/CsPbBr_3_-1 (T/C-1), TiO_2_/CsPbBr_3_-3 (T/C-3) and CsPbBr_3_. **b** The FTIR spectrum of the TiO_2_, TiO_2_/CsPbBr_3_-1, TiO_2_/CsPbBr_3_-3 and CsPbBr_3_. **c** The TG curve of the TiO_2_, TiO_2_/CsPbBr_3_-1, TiO_2_/CsPbBr_3_-3 and CsPbBr_3_

The structure of fluorescent electrophoretic particles was investigated by XRD (X-ray diffraction), FTIR (Fourier transform infrared spectroscopy), and TG (Thermogravimetric analysis) measurements. The rutile TiO_2_ particle is consistent with PDF card #21-1276, and orthorhombic CsPbBr_3_ is consistent with PDF card #18-0364, as shown in Fig. 2a. The TiO_2_/CsPbBr_3_-3 particles exhibit the strong TiO_2_ characteristic diffraction peak. As can be seen from the enlarged plots regions (i) at 20°-25° and (ii) at 28°-32°, the (110) peak at 21.5° and the (002) peak at 30.7° belong to the CsPbBr_3_. The CsPbBr_3_’s characteristic peak intensity of fluorescent electrophoretic particles increases as the amount of CsPbBr_3_ increases, demonstrating the CsPbBr_3_ efficiently loading onto the TiO_2_ particles^38^. Moreover, the XRD curves of pure TiO_2_, TiO_2_ after silane coupling, and TiO_2_ after polymerization are shown in Figure S2. The XRD peaks of all three TiO_2_ particles exhibit almost the same, indicating that silane coupling and polymerization would not affect the crystal structure of TiO_2_ particles.

In addition, we tested the FTIR spectra to verify the structure of the fluorescent electrophoretic particles, as exhibited in Fig. 2b. The absorption peaks around 700 cm^-1^ are the Ti-O bonds of TiO_2_, and the absorption peaks at 2750-3000 cm^-1^ is -CH_3_ vibration bonds from OAm as well as the acrylic in the CsPbBr_3_^39,40^. For the TiO_2_/CsPbBr_3_-3 particles, the preparation method is similar with CsPbBr_3_, so it contains Ti-O bonds from the TiO_2_ and -CH_3_ vibration bonds from the OAm on the CsPbBr_3_ surface, respectively^39,40^. The FTIR spectra further confirms that CsPbBr_3_ successfully loaded on TiO_2_ particles. To further explore the content of CsPbBr_3_ loading, we tested the TG curves, as shown in Fig. 2c. Pure TiO_2_ loses a 3% mass ratio originating from the silane coupling agent after 900 °C^24^. Compared with TiO_2_, the TiO_2_/CsPbBr_3_-3 loses a 1% mass ratio after 900 °C. Besides, the CPB loses a 99% mass ratio after 900 °C due to the thermal decomposition. Hence, it can be considered that nearly 1% of the mass of the QDs was loaded on the TiO_2_ particles.

**Fig. 3:**
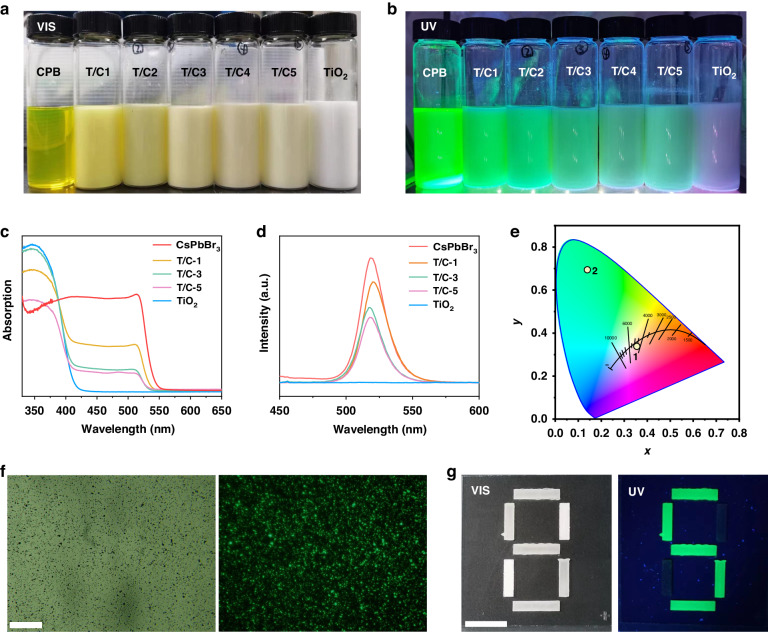
Optical properties of fluorescent electrophoretic particles. **a, b** The photograph of the fluorescent electrophoretic particles with different ratio of CPB under visible and UV light. **c** The absorption spectrum of the fluorescent electrophoretic particles. **d** The PL emission spectrum of the fluorescent electrophoretic particles. **e** The CIE chromaticity diagram of the TiO_2_/CsPbBr_3_-3 at white and green state under ambient light and 365 nm UV light. **f** The fluorescence microscopy of the fluorescent electrophoretic particles TiO_2_/CsPbBr_3_-3. Scale bar: 100 μm. **g** The AC application of the fluorescent electrophoretic particles TiO_2_/CsPbBr_3_-3. Scale bar: 2 cm

To investigate the optical properties of the fluorescent electrophoretic particles, we have tested the whiteness, UV absorption spectra, fluorescence spectrum, and fluorescence microscopy. The photograph of fluorescent electrophoretic particles with different CPB concentrations are shown in Fig. 3a,b. Pure CPB is the transparent yellow-green solution, and pure TiO_2_ is a white solution. As the content of CPB decreases, the solution gradually changes from yellow to pure white under ambient light. With the increase of TiO_2_ content, the bright fluorescence intensity slowly decreased under UV light. The absorption spectra and Tauc plot curves of the fluorescent electrophoretic particles are shown in Fig. 3c and S3. The absorption wavelength of CPB is 550 nm, and the absorption wavelength of TiO_2_ is 400 nm, corresponding band gaps of 2.33 eV and 3.10 eV, respectively. For the fluorescent electrophoretic particles TiO_2_/CsPbBr_3_-3, both the absorption peaks of TiO_2_ and CPB were observed. The absorption intensity at 400 nm of the fluorescent electrophoretic particles became stronger with the increase of TiO_2_ content. Noticeably, the non-continuous modulation of the absorption spectra indicates that the TiO_2_ particles are in contact and interact with CPB^39,41^.

Moreover, the CPB has a sharp and single PL (Photoluminescence spectroscopy) emission peak at 520 nm, as shown in Fig. 3d. The intensity of PL decreases significantly with an increase of TiO_2_ content until no PL emission of pure TiO_2_ particles. The excitation of the PL spectra is 380 nm, and the full-width-at-half-maximum (FWHM) of TiO_2_/CsPbBr_3_-3 is about 20 nm, exhibiting good monochromaticity. In addition, the whiteness and PL intensity of fluorescent electrophoretic particles is shown in Figure S4, where TiO_2_ exhibits a high whiteness of about 85. As the CPB content increases, the whiteness of electrophoretic particles gradually decreases until it drops to a pure CPB of 50. To balance the whiteness and PL emission intensity, we choose TiO_2_/CsPbBr_3_-3 as fluorescent electrophoretic particles. The fluorescent lifetime curves and the fitting results of the fluorescent electrophoretic particles are shown in Figure S6 and Table S1. Compared with the lifetime of 26 ns for pure CPB, the TiO_2_/CsPbBr_3_-3 has a faster lifetime of 17.1 ns, demonstrating that the fluorescent electrophoretic particles TiO_2_/CsPbBr_3_-3 have a faster charge-transfer process^42^. The shorter lifetime indicates that a part of the electrons in the excited state of the CPB would transfer to the surface of the TiO_2_ particles. After the electrons transfer to the TiO_2_, the time of electrons stay in the excited state would become shorter, resulting in a faster rate of attenuation of fluorescence intensity, which is consistent with the decrease in fluorescence intensity.

The CIE coordinates of the TiO_2_/CsPbBr_3_-3 are (0.3588, 0.3372) and (0.1334, 0.7342) for the white and green states, as present in Fig. 3e. The long-distance between the green state and white state is beneficial for the application of fluorescent electrophoretic particles in AC devices. To further explore the optical properties of fluorescent electrophoretic particles, we have tested the fluorescence microscope. The TiO_2_ blank sample doesn’t emit green fluorescence in the fluorescence microscope, as exhibited in Figure S5. In contrast, the fluorescence microscopy of TiO_2_/CsPbBr_3_-3 is shown in Fig. 3f, and all the particles can emit green fluorescence well, which indicates that the CPB can uniformly load onto TiO_2_. It can be seen from the absorption spectrum and PL spectra that TiO_2_ has strong absorption of UV light, so TiO_2_ particles would exhibit a black state under UV light. To verify the potential application of fluorescent electrophoretic particles in the AC device, we coated the number “5” pattern with fluorescent electrophoretic particles in the number “8” pattern, while the other places coated with normal white electrophoretic particles. The pattern shows the number “8” under visible light, while the secure number “5” pattern shows bright green light under UV light, as shown in Fig. 3g. The results demonstrate that fluorescent electrophoretic particles have a good potential application in AC devices and document security.

**Fig. 4:**
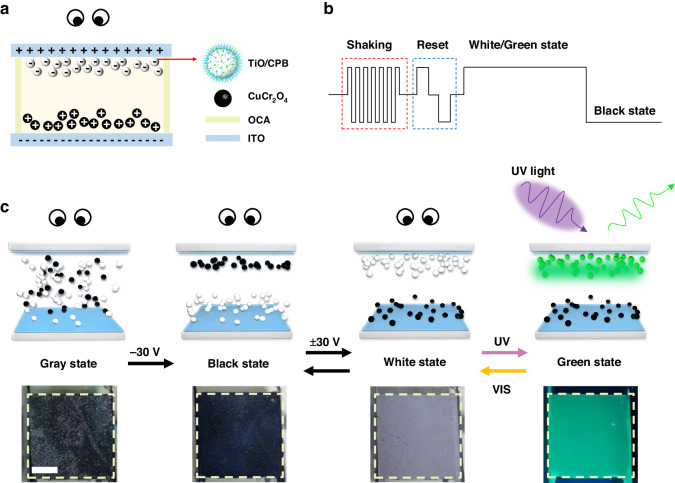
The schematic diagram and photograph of fluorescent EPD. **a** The schematic diagram of the fluorescent EPD. **b** The waveform of the fluorescent EPD. **c** The schematic diagram and photograph of the fluorescent EPD at white, green and black state. Scale bar: 1 cm

The electrophoretic dispersion solution was prepared by mixing the fluorescent electrophoretic particles, including negatively charged white fluorescent electrophoretic particles, positively charged black particles (CrCu_2_O_4_), solvent Isopar G, thickener, and CCA additives. Then, the fluorescent EPD was fabricated, including the electrophoretic dispersion solution, upper and bottom ITO electrodes, and 40 μm OCA as a spacer layer, as shown in Fig. 4a. When the upper electrode is at positive voltage and the bottom electrode is at negative voltage, the fluorescent electrophoretic particles would be driven to the upper electrode and reflective the light. Then, the device exhibits a white state under visual light. On the country, the fluorescent EPD absorbs the ambient light and exhibits the black state.

The driving waveform of the EPD is shown in Fig. 4b. Firstly, the shaking waveform is used to activate the electrophoretic particles, leading the CCA to be adequately charged with the electrophoretic particles^20^. Then, the DC balance waveform is used to eliminate the built-in electric field between the particles, thus increasing the stability of the particles^43^. Lastly, the positive voltage is given to achieve the white and green states of the device under the switch of UV light, and the negative voltage is given to achieve the black state. The schematic and photograph of the EPD are shown in Fig. 4c, and the black and white states of the EPD are switched by ±30 V voltage under ambient light. Besides, the white and green states of the EPD are switched by UV light, which can excite the fluorescent electrophoretic particles and emit the green fluorescence. The fluorescent EPD can multifunctional display under the switching of electric field and UV light. Multifunctional display facilitates the fluorescent EPD in dynamic AC devices, and the bright green fluorescence intensity of fluorescent EPD is also beneficial to achieve the color EPD.

To further evaluate the performance of the EPD, we have tested the contrast ratio, threshold voltage, response time, and long-term stability of the fluorescent EPD. The contrast ratio of EPD can be calculated by white reflectance divided the black reflectance (*R*_white_/ *R*_black_). The whiteness of the fluorescent EPD is increase with the content of TiO_2_, and then optimize the contrast ratio of the TiO/CPB-3 EPD reaches 17 and exhibit the good display performance, as shown in Figure S7. In addition, we tested the ambient contrast ratio (ACR) of the fluorescent electrophoretic display device between the green and white states at different ambient light illuminance, as shown in Figure S8. When the fluorescent EPD is in the lower white light illumination, the device has a higher ACR. The ACR value of fluorescent EPD is 569 under the 0.1 lux ambient light, and the green light intensity would be higher in the darker environments. As the ambient light illuminance increases, the ACR of the fluorescent EPD would decrease, which is consistent with the results of self-luminous display^44^. When the illuminance of ambient light increases to 2000 lux, the ACR of the EPD between the green and white states is close to 1, which indicates that the anti-counterfeiting effect decreases at illuminances higher than 2000 lux. Generally, the illuminance of an indoor environment and cloudy outdoor without direct sunlight is less than 500 lux and 2000 lux, respectively, so the anti-counterfeiting devices based on fluorescent EPD are suitable for daily life and working environments. When the ambient light illuminance is too high, we can use the normal reflective mode (black-and-white state) of the fluorescent EPD for display^45^. Combining the advantages of the self-luminous and reflective display, the fluorescent EPD can normally work under the different ambient light illuminance environments.

**Fig. 5:**
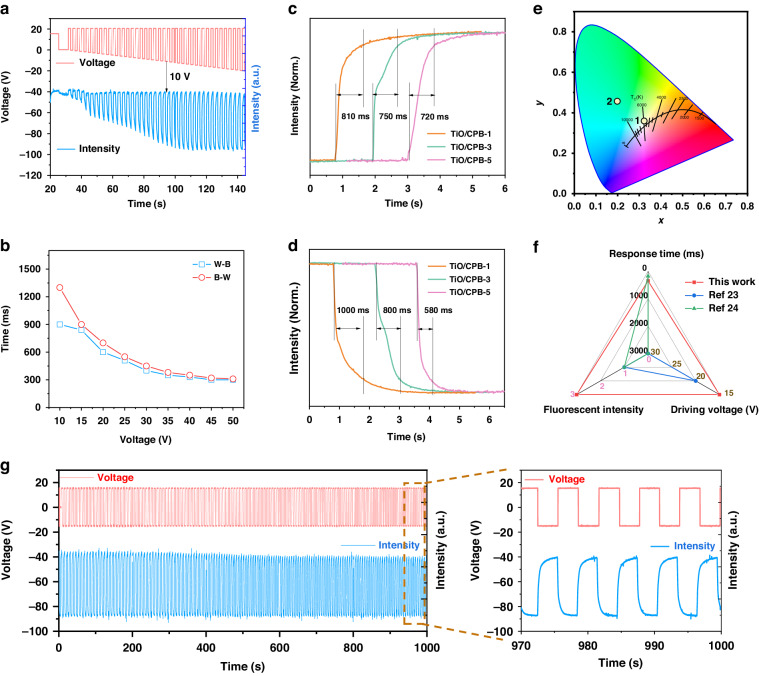
The optoelectronic performance of fluorescent EPD. **a** The threshold voltage test of the fluorescent EPD from the black to white state. **b** The response time of the fluorescent EPD from white to black state and black to white state under different driving voltage. **c** The response time curve of the fluorescent EPD from white to black state with different ratio of CPB under 15 V. **d** The response time curve of the fluorescent EPD from black to white state with different ratio of CPB under 15 V. **e** The CIE chromaticity diagram of the fluorescent EPD at white and green state under ambient light and 365 nm UV light. **f** A comprehensive comparison of our fluorescent EPD with other reported. **g** Long-time stability cycles test of fluorescent EPD in 1000 s

The schematic diagram of the electro-optical response test platform of the EPD is shown in Figure S9. The white light penetrates the L1 lens and then illuminates the EPD. Then, EPD would reflect light which penetrates the L2 lens to be detected by the detector. The electro-optical response test platform provides an important test method for the response time test, threshold voltage, and stability test. The threshold voltage test can provide a guide for the driving waveform of the fluorescent EPD. The electrophoretic particles start to move at 5 V, while the response speed is too slow at the low voltage, resulting in the particles not returning to the initial position, as shown in Fig. 5a and S10. When the driving voltage is increased to 10 V, the electrophoretic particles can return to the initial position. Hence, the device can be driven when the driving voltage is larger than 10 V.

The response time of the fluorescent EPD from white to black state (WTB) and black to white state (BTW) under different driving voltage is shown in Fig. 5b. The response time of the EPD from WTB is 1300 ms under 10 V. As the driving voltage increases, the response speed of the EPD decreases to 300 ms due to the increase of electronic field intensity and achieves a fast fluorescent EPD response. The response time of TiO/CPB-3 is 350 ms and 450 ms for WTB and BTW under 30 V, presented in Figure S11. The response time curves of fluorescent EPD with different ratios of CPB from white to black state are shown in Fig. 5c. With the increase of TiO_2_ content, the response time of fluorescent EPD decrease from 810 ms to 720 ms for TiO/CPB-1 to TiO/CPB-5 under 15 V. Besides, the response time decrease from 1000 ms to 580 ms for TiO/CPB-1 to TiO/CPB-5 from black to white state under 15 V. The faster response speed of TiO/CPB-5 probably attributed to increase the content of TiO_2_ and charged functional groups, which increasing the surface zeta potential of the particle and response speed of the EPD.

The PL spectrum of the fluorescent EPD at green state is shown in Figure S12, exhibiting a single emission peak at 500 nm, indicating that fluorescent EPDs have a good fluorescent luminescence. In addition, the CIE coordinates diagram of the fluorescent EPD are (0.3232, 0.3639) and (0.1979, 0.4607) for the white and green states, as present in Fig. 5e. A comprehensive comparison of our fluorescent EPD with other works is presented in Fig. 5f, illustrating that our device has a lower driving voltage of 15 V and stronger fluorescence intensity. In terms of devices, our fluorescent EPD exhibits a fast response time of 350 ms, high white and black state contrast ratio of 17, and high intensity of green fluorescence. Then, we tested the long-term stability of the device, which was switched between the black and white states in 1000 s, as exhibited in Fig. 5g. The intensity of the device stayed almost unchanged after long-term driving, indicating that the device has good stability, which is beneficial to AC devices.

**Fig. 6:**
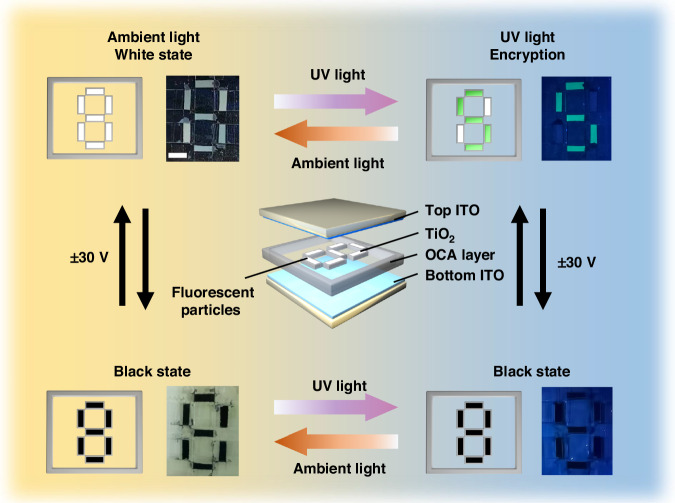
The AC application based on the fluorescent EPD. Scale bar: 1 cm

To extend the fluorescent EPD in AC applications, we have fabricated a multifunctional AC device that can realize multimode display under the switching of electric field and UV light. The structure of the AC device is shown in Fig. 6, which consists of a sandwich structure (top and bottom ITO layer, patterned OCA with number “8”, which can be filled with electrophoretic dispersion). In addition, we filled the number “5” pattern with fluorescent electrophoretic dispersion in the number “8” pattern, while the other two places were filled with normal electrophoretic dispersion. In visible light, the EPD can achieve the black and white number “8” pattern switched under 30 V. When the white particles are driven to the top ITO layer, the green state of the number “5” pattern and white state of number “8” pattern can be switched by switching the UV light. It is worth noting that when the EPD is in the black state, the black number “8” is exhibited under ambient light and no pattern in UV light. The EPD achieve the dynamic AC and multifunctional display, which can effectively develop the security. By controlling the electric field and UV light, the fluorescent EPD can hide confidential information and achieve privacy controlled, intelligent, and multifunctional AC devices. The color of the pattern can be switched by controlling the voltage, so it is able to achieve dynamic anti-counterfeiting, and the device has relatively higher confidentiality. Considering the commercial application, future research work can be equipped with a commercial PM (passive matrix) or AM (active matrix) driving circuit to realize the pattern switching of an anti-counterfeiting device, which can make the fluorescent EPD suitable for more usage scenarios.

## Discussion

In conclusion, we loaded CsPbBr_3_ QD on the white TiO_2_ particles and further polymerized them to obtain fluorescent electrophoretic particles TiO/CPB-3. The particles showed a white state under ambient light and a green state under UV light, exhibited an ideal AC effect. Then, we fabricated the fluorescent EPD based on the fluorescent electrophoretic particles TiO/CPB-3, which exhibited a fast response time of 350 ms and a bright green fluorescence state under UV light. Finally, we fabricated a multifunctional AC device based on fluorescent EPD, which can achieve a multimode and dynamic AC display by switching the electric field and UV light. This work demonstrates that the fluorescent electrophoretic particles have a great potential for multifunctional AC devices.

## Materials and methods

### Materials

All reagents were of analytical grade and used as received without further purification. Lead bromide (PbBr_2_ 99%), Caesium bromide (CsBr 99.9%), Oleic acid (OA), Oleylamine (OAm), 2,2’-Azobisisoheptonitrile (ABVN), 3-(Trimethoxysilyl) propyl methacrylate (MPS, KH570), lauryl methacrylate (LMA), Acetic acid, sodium dodecyl benzene sulfonate (SDBS), hexane, and N, N-Dimethylformamide (DMF) were purchased from Shanghai Aladdin Biochemical Technology Corp., Ltd. Pure TiO_2_ particles was purchased from the YuMu New materials Corp., Ltd. Methylacrylic acid (MAA), Ethyl acetate (EA) and Methacryloxypropyl-terminated polydimethylsiloxane (stabilizer) was purchased from Shanghai Macklin Biochemical Corp., Ltd. Toluene was purchased from Guangzhou Chemical Reagent Factory. Non-polar solvent Isopar G was purchased from Shanghai Tichem Chemical Co., Ltd. The charge control agent, thickener, and black particles were supplied from Guangzhou OED Technologies Co., Ltd. ITO glass electrode was purchased from South China Science & Technology Co., Ltd.

### Synthesis of TiO_2_ silane particles

5 g TiO_2_, 5 g MPS, 0.5 mL Acetic acid, 250 mg SDBS, and 50 mL toluene were ball milled at 400 rpm for 5 hours. Then, the TiO_2_-MPS particles were obtained by centrifugation, washed with toluene several times, and dried in the oven at 60 °C for 6 h.

### Synthesis of TiO_2_/CsPbBr_3_ (T/C) particles

21 mg CsBr, 73.4 mg PbBr_2_ were stirred and dissolved in 5 mL DMF. Then, 80 μL OAm and 150 μL MAA were added into the above solution and stirred. Next, 300 μL of the above solution was quickly injected into the 20 mL toluene and green colloidal CsPbBr_3_/toluene solution was obtained. After that, the CsPbBr_3_ NCs were washed with EA and n-hexane, and dried at 40 °C in a vacuum drying oven. The different ratio of TiO_2_/CsPbBr_3_ particles was synthesized by adding the 300 μL perovskite precursor solution into the 20 mL toluene solution with different mass of TiO_2_ particles.

### Polymerization TiO_2_/CsPbBr_3_-PLMA (TiO/CPB) particles

100 mg TiO_2_/CsPbBr_3_ particles, 300 mg LMA, 50 μL stabilizer, and 20 mL Isopar G solvent were added into the four-necked flask at 40 °C for 1 h under N_2_. Then, 20 mg ABVN was dissolved in the 10 mL Isopar G solvent, and the mixture solution were dropped slowly into the four-necked flask by constant pressure separatory funnel. The TiO/CPB particles were obtained after 5 h polymerization. Next, the TiO/CPB particles were obtained by washing with Isopar G solvent several times and drying in the oven at 40 °C for 6 h. The white inks were further obtained by dispersed the 100 mg TiO/CPB particles in to the 500 mg Isopar G.

### Preparation of the electrophoretic dispersion

The electrophoretic dispersion consisted of 250 mg white inks, 60 mg black inks, 20 mg CCA, and 20 mg thickener. Before filling the electrophoretic dispersion into the electrophoretic test cell, the electrophoretic dispersion was stirred for 12 h at 100 rpm.

### Preparation of the electrophoretic test cell

The ITO electrode was cleaned under the UV/Ozone 20 min treatment. The electrophoretic test cell was fabricated by two ITO electrode, and the 40 μm thickness of cell was controlled by 40 μm OCA. Then, the electrophoretic dispersion was filled into the test cell by capillary force. After filled the electrophoretic dispersion into the electrophoretic test cell, the ultraviolet (UV) adhesive (NOA 65, provided by Norland) sealed the two sides of the cell to test the optoelectronic response performance.

### Characterization

The structures of TiO_2_ and perovskite were recorded by X-ray diffraction (XRD) (Empyrean) at 40 kV and 40 mA (Cu Kα X-ray radiation source) with scanning speed and step interval of 4°·min^-1^ and 0.02°. The UV-visible absorption spectrum was measured by a UV-visible spectrophotometer (Lambda 950) ranging from 400 to 800 nm. The PL emission spectrum and time-resolved fluorescence decay curves were performed by a fluorescence spectrometer (FLS 980, Edinburgh). The contact angle of the fluorescent electrophoretic particles was measured by the contact angle meter (DataPhysics OCA 15EC). DLS curve and zeta potential were test by the light scattering spectrophotometer (Malvern Zetasizer). Fluorescence microscopy images were obtained by fluorescence microscope (MF 52, Guangzhou Microshot Optical Technology Co. LTD). Fourier transform infrared spectroscopy (FTIR) (VERTEX 70 V spectrometer, Bruker) was measured in the spectral region from 400 to 4000 cm^–1^ with KBr as a beam splitter. Thermogravimetric curves were characterized by a thermogravimetric analyzer (TG209F1 libra). The whiteness and contrast ratio of EPD was measured by the Eye-One spectrophotometer (X-Rite). The electro-optical response curve of fluorescent EPD was detected by a reflective light photodetector as shown in Figure S9. A special designed driving waveform was applied to the test the fluorescent EPD by the LabVIEW system. The photodetector (DH-GDT) would collect the reflect light and show the intensity.

### Supplementary information

Supplemental Material
