# Supplementary material for: Fluorescent, multifunctional anti-counterfeiting, fast response electrophoretic display based on TiO2/CsPbBr3 composite particles
Source: Light Sci Appl. 2024 Aug 20;13:198. doi: 10.1038/s41377-024-01526-x (PMC11335904; doi:10.1038/s41377-024-01526-x)
Supplement: Supplementary file 1 — Supplemental Material [file 41377_2024_1526_MOESM1_ESM.docx]

**Supplementary Information for**

**Fluorescent, multifunctional anticounterfeiting,** **fast response electrophoretic display based on TiO_2_/CsPbBr_3_ composite particles**

Guangyou Liu, Xinzao Wu, Feng Xiong, Jinglan Yang, Yunhe Liu, Jie Liu,

Zhuohang Li, Zong Qin, Shaozhi Deng and Bo-Ru Yang*.

1 State Key Laboratory of Opto-electronic Materials and Technology, Guangdong Province Key Laboratory of Display Materials and Technologies, School of Electronics and Information Technology, Sun Yat-Sen University, Guangzhou 510006, China

* Email: paulyang68@me.com


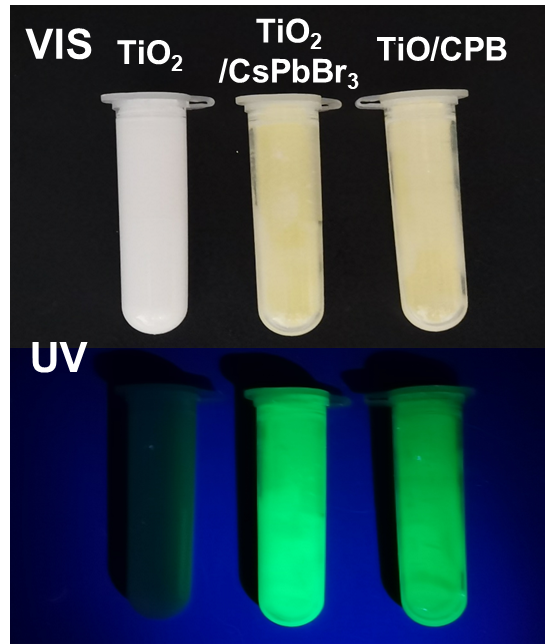


Figure S1. The photograph of TiO_2_, TiO_2_/CsPbBr_3_, TiO_2_/CsPbBr_3_-PLMA (TiO/CPB) under the visual light and UV light.

Figure S2. The XRD curves of TiO_2_, TiO_2_-MPS, TiO_2_-MPS-PLMA.

Figure S3. The Tauc plot based on the UV−vis absorption spectra of the fluorescent electrophoretic particles.

Figure S4. The whiteness and PL intensity of the fluorescent electrophoretic particles.


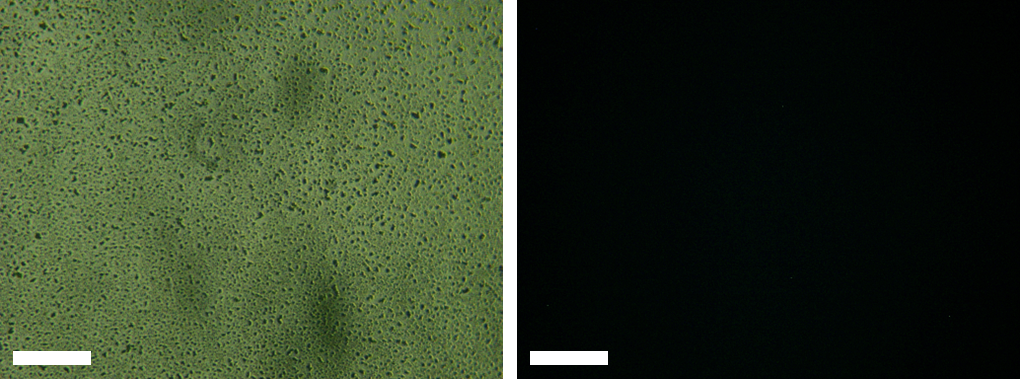


Figure S5. The fluorescence microscope of the TiO_2_, (scale bar is 100 μm).

**Table S1.** Fitted parameters of the PL decay curves for the fluorescent electrophoretic particles.

|  | *A*_1_ (%) | *τ*_1_ (ns) | *A*_2_ (%) | *τ*_2_ (ns) | *τ*_av_ (ns) |
| --- | --- | --- | --- | --- | --- |
| CsPbBr_3_ | 95.4 | 10.97 | 4.6 | 72.95 | 26 |
| TiO_2_/CsPbBr_3_-1 | 95.3 | 10.5 | 4.7 | 67.3 | 24.1 |
| TiO_2_/CsPbBr_3_-3 | 96.2 | 9.29 | 3.8 | 52.22 | 17.1 |
| TiO_2_/CsPbBr_3_-5 | 97.4 | 7.64 | 2.6 | 48.97 | 13.7 |

The average PL lifetime (*τ*_ave_) can be calculated according to the formula S1

(S1)

$$\tau_{\mathrm{ave}}=\frac{{(\tau}_{1}^{2}*A_{1}+\tau_{2}^{2}*A_{2}\text{ )}}{\text{(}\tau_{1}*A_{1}+\tau_{2}*A_{2}\text{ ) }}$$


Figure S6. The lifetime spectra of the fluorescent electrophoretic particles.

Figure S7. The white/black state reflectance and the contrast ratio of the fluorescent EPD.

The ambient contrast ratio (ACR) of the fluorescent EPD between the green and white states is calculated as shown in formula S2, where *L*_green_ is the luminance of the emitted light in the green state, *L*^’^_white_ is the luminance of the device which exhibited white state under the UV light source and external ambient white light source, and *L*_white_ is the luminance of the device which exhibited white state under ambient white light source.

$$\begin{aligned} ACR=\frac{L_{\mathrm{on}}}{L_{\mathrm{off}}}=\frac{L_{\mathrm{green}}+L_{\mathrm{white}}^{'}}{L_{\mathrm{white}}}\#\left( S2 \right) \end{aligned}$$


Figure S8. The ACR curve of the fluorescent electrophoretic particles under the different ambient light illuminance.


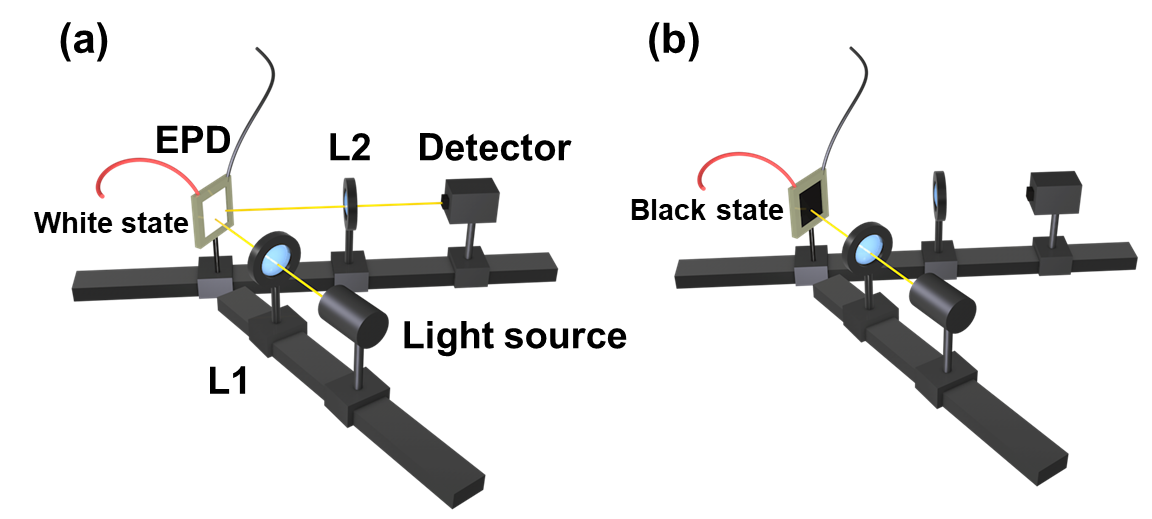


Figure S9. The electro-optical response test platform of the fluorescent EPD (a) EPD at white state (b) EPD at black state.

Figure S10. The threshold voltage test of the fluorescent EPD from white to black state.


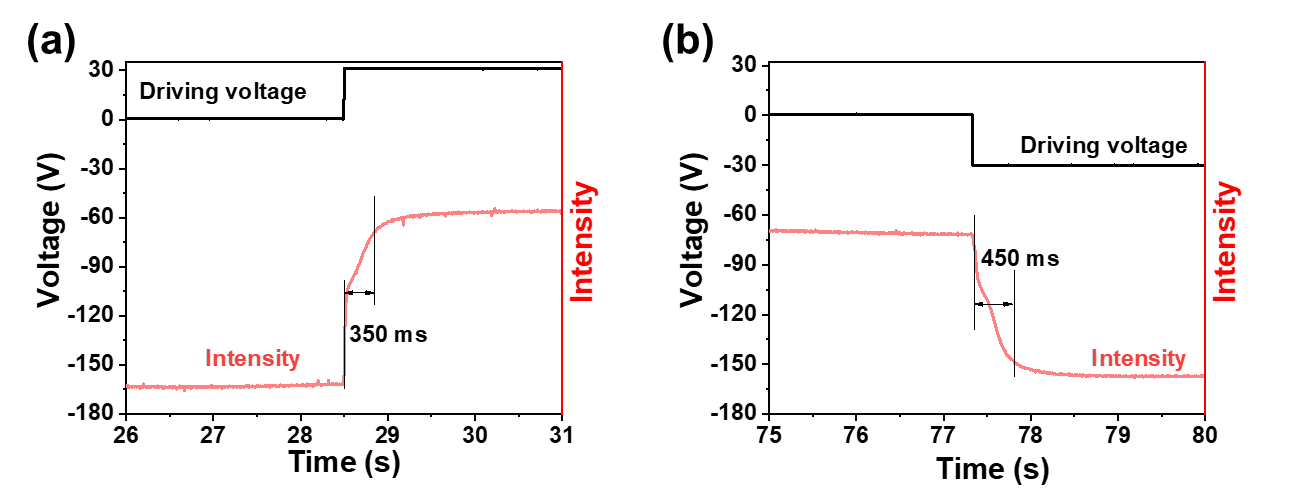


Figure S11. (a) The response time curve of the fluorescent EPD from white to black state of TiO/CPB-3 under 30 V. (b) The response time curve of the fluorescent EPD from black to white state of TiO/CPB-3 under 30 V.

Figure S12. The PL spectrum of the fluorescent EPD at green state.
